# Transcriptome profiling of two rice varieties reveals their molecular responses under high night-time temperature

**DOI:** 10.1371/journal.pone.0311746

**Published:** 2024-10-10

**Authors:** Wardah K. Mustahsan, Yuya Liang, Abdul R. Mohammed, Charles D. Johnson, Endang M. Septiningsih, Lee Tarpley, Michael J. Thomson

**Affiliations:** 1 Department of Soil and Crop Sciences, Texas A&M University, College Station, Texas, Unted States of America; 2 Texas A&M Agrilife Research & Extension Center, Beaumont, Texas, Unted States of America; 3 Genomics and Bioinformatics Service, Texas A&M AgriLife Research, College Station, Texas, Unted States of America; Shiraz University, ISLAMIC REPUBLIC OF IRAN

## Abstract

High night-time temperatures (HNT) pose a threat to the sustainability of crop production, including rice. HNT can affect crop productivity and quality by influencing plant physiology, morphology, and phenology. The ethylene perception inhibitor, 1-methylcyclopropene (1-MCP), can minimize HNT-induced damage to plant membranes, thereby preventing decrease in rice yield. In this study, we employed a transcriptome approach to investigate the effects of HNT, 1-MCP, and their interaction on two Texas rice varieties, Antonio and Colorado. The plants were exposed to temperatures of 25°C (ambient night-time temperature, ANT) and 30°C (HNT) using an infrared heating system from the booting stage until harvest, while 1-MCP was applied at the booting stage of rice development. Several physiological and agronomical traits were evaluated under each condition to assess plant responses. Leaf tissues were collected from the plants grown in the ANT and HNT conditions after the heat stress and 1-MCP treatments. Based on agronomic performance, Colorado was less negatively affected than Antonio under HNT, showing a slight reduction in spikelet fertility and leaf photosynthetic rate but no significant reduction in yield. The application of 1-MCP significantly mitigated the adverse effects of HNT in Antonio. However, no significant differences were observed in yield and leaf photosynthetic rate in Colorado. Furthermore, transcriptomic data revealed distinct responsive mechanisms in Antonio and Colorado in response to both HNT and 1-MCP. Several ethylene and senescence-related transcription factors (TFs) were identified only in Antonio, suggesting that 1-MCP affected the ethylene signaling pathway in Antonio but not in Colorado. These findings contribute to our understanding of the physiological differences between varieties exhibiting susceptible and tolerant responses to high night-time temperatures, as well as their response to 1-MCP and ethylene regulation under 1-MCP.

## Introduction

Rice (*Oryza sativa* L.) is one of the most essential grains produced worldwide. It serves as a staple food for more than 3 billion people in different countries around the world. However, due to the changes in the climate today, crop production faces great challenges, such as increased drought and heat stress. High night-time temperature (HNT) has become a detrimental factor limiting rice yield and grain quality globally [1–4]. Grain yields declined by 10% for each 1°C increase in the minimum (i.e. night) temperature during the dry season, whereas the effect of maximum temperature on crop yield was insignificant [5]. The mid-south US is vulnerable to periods of HNT, which can decrease rice fertility and yield [6]. HNT stress is also known to increase oxidative stress and ethylene levels in plants which can induce the production of ethylene-triggered reactive oxygen species (ROS) [7]. ROS may cause DNA, protein, and membrane damage [8, 9], which can affect various variables, including leaky membranes, which affect the production, consumption, and transfer of photosynthates. These physiological responses can have negative effects on grain quality and grain yield. Ethylene is a multifunctional plant hormone, which not only raises signals for stress responses but also affects growth and development [10]. The accumulation of ethylene in the plant during heat stress accelerates senescence and leads to reduced spikelet fertility and grain yield [11–13]. 1-methylcyclopropene (1-MCP) is an ethylene perception inhibitor that inhibits the production of ROS and minimizes HNT stress-induced damage. By competitively binding to the ethylene receptor, 1-MCP prevents the chemical effects of ethylene, thereby preventing yield loss, and leads to an improvement in yield-related traits of crops such as rice and soybean [6, 7, 14]. Comprehensive analysis of the molecular mechanisms involved in the relationship between HNT and 1-MCP will shed a light on the mechanisms of tolerance and may provide opportunities for genetic manipulation for more effective approaches to overcome the challenges presented due to HNT stress.

Whole-genome transcriptomic studies are often used to study molecular mechanisms of various plant characteristics under different growth conditions and have been applied in many crops, including rice, under diverse abiotic stress conditions [15–19]. Several transcriptomic studies have demonstrated the significance of breeding rice towards improving thermotolerance. For example, [20] investigated the effects of heat stress on panicle development in two rice cultivars exposed to different heat stress conditions (40°C vs. 32°C). Their findings revealed that high temperature conditions triggered the signaling of endogenous hormones, promoting heat tolerance, but also negatively regulated starch and sucrose metabolism, thus impeding panicle development. A study by [21] examined the impact of heat stress on anther response during anthesis in rice and identified several key gene categories associated with anther heat tolerance, including nucleic acid, protein metabolism, and transcription factors. In another study by [22], contrasting rice varieties were subjected to heat stress conditions (37°C and 42°C), highlighting the crucial roles of auxin and ABA response genes in heat tolerance. Additionally, the study explored the interaction between these genes and transcription factors (e.g., HSF, NAM, ATAF and CUC (NAC), and WRKY). However, these studies did not investigate the role of high night-time temperature and interactions with ethylene perception.

The aim of the current study was to investigate the underlying molecular mechanisms involved with HNT conditions, the impact of application of 1-MCP, and the interaction between the two factors. RNA-seq was performed on two rice varieties, ‘Colorado’ and ‘Antonio’, having different responses to HNT. Additionally, several agronomical and physiological traits were measured. Our results of both transcriptome profiling and phenotypic data concurred that Colorado was more tolerant under HNT stress. The application of 1-MCP largely increased the yield, spikelet fertility, and leaf photosynthetic rate in HNT susceptible variety, Antonio. On the other hand, 1-MCP did not show beneficial effects to Colorado under HNT. Some of the differentially expressed genes (DEGs) detected in this study can be further investigated through further molecular studies and manipulation for crop improvement to enhance plant performance and mitigate grain yield reduction under HNT conditions.

## Materials and methods

### Plant materials, heat treatment and 1-MCP treatment

Colorado and Antonio are two elite long-grain rice varieties [23, 24] selected for this study due to their differing performances in agronomic traits under high nighttime temperature (HNT) conditions, such as yield. Colorado is a high-yielding rice cultivar derived from ‘Cocodrie’ and ‘L202’, developed for its shortened growing cycle and lower water usage. On the other hand, Antonio, derived from a cross of ‘Cypress’ and ‘Cocodrie’, was developed for early maturation. Plant materials were acquired from the Texas A&M AgriLife Research Center at Beaumont, TX. For each variety, there were four conditions with four replicates for the phenotyping data and three replicates for the RNA extraction and sequencing: (1) 25°C ambient night-time temperature (ANT; greenhouse settings) with 1-MCP, (2) 25°C ANT without 1-MCP, (3) 30°C HNT with 1-MCP, and (4) 30°C HNT without 1-MCP. In total there were 32 plant samples evaluated for phenotyping using the two varieties Colorado and Antonio in this study. From those 32 samples, 24 samples in total were used for RNA-Seq analysis.

The plants were grown in 3-liter pots filled with clay soil, which was typical of the rice farms in the area, using a randomized complete block design (RCBD) in the greenhouse at the Texas A&M AgriLife Beaumont Research & Extension Center in 2017. Each pot had five seeds placed at a depth of 2.5 cm. Once the seedlings emerged, the plants were thinned down to one plant per pot. All the plants were maintained at ANT until the booting stage. At booting stage, half of the plants were selected randomly and moved under heat lamps (HNT: 30°C) which were positioned 1.0 m above the topmost part of the plants and provided controlled infrared radiation enrichment [6]. The remaining half of the plants remained at ambient conditions which were setup with dummy heaters that provided a similarly shaded environment. Within each temperature condition, half of the plants were randomly selected to be treated with 1-MCP (courtesy of AgroFresh, Pennsylvania, U.S.A.) at the booting stage. High night-time temperature conditions (from 8:00 PM to 6:00 AM) were imposed starting at the booting stage and maintained until harvest. The temperature treatments in the greenhouse were monitored independently of the temperature-control system through the use of standalone sensor/loggers (HOBO, H08-003-02; Onset Computer Corporation, Bourne, MA, USA), which were placed a few centimeters into the canopy in both portions of the study.

### Phenotypic evaluation

The physiological parameters measured at physiological maturity were grain yield and spikelet fertility. Leaf photosynthetic rate was measured five days after treatment. Spikelet fertility was calculated from filled and unfilled grain numbers on each of the selected panicles. All panicles in each pot were manually threshed and manually separated as filled and unfilled grains. Grain yield was measured as the total grain weight per pot. In our study, leaf net photosynthetic rate (Pn) was measured as previously reported by [6]. In brief, Pn was measured on the penultimate leaves using a LI-6400 portable photosynthesis system (LI-COR Inc., Lincoln, NE, USA) between 10:00 AM and 12:00 PM. The photosynthetic photon flux density (PPFD) was set to 1500 μmol m^−2^ s^−1^. The temperature and CO_2_ concentration in the leaf cuvette were set to 25°C and 360 ppm (ambient CO_2_ concentration in the greenhouse), respectively. Humidity in the cuvette was controlled by circulation of the air through desiccant. A steady flow rate of 500 μmol s^−1^ was maintained in the leaf chamber. Three individual leaves (penultimate position) per plant were measured. The student’s *t*-test was performed using JMP Pro 12.2 to determine the significance of difference for all traits.

### RNA extraction and sequencing

A total of 24 leaf tissue samples were collected from Antonio and Colorado. For each temperature condition and chemical treatment, three biological replicates were collected. These samples were collected at 5 days post exposure to night-time temperature treatments (HNT/ANT), at booting stage, and flash frozen in liquid nitrogen. The RNA extraction procedure was performed using the QIAgen RNeasy Plant Mini kit. These RNA samples were submitted to Texas A&M AgriLife Genomics and Bioinformatics Service (TxGen; College Station, TX, USA) for RNA-Seq library preparation and sequencing. The libraries were run on multiple lanes of an Illumina HiSeq 4000 (San Diego, CA, USA) to provide at least 25 million reads (75 nt pair-end) per sample.

### Data processing

In total, 24 cDNA libraries were submitted for sequencing, including three biological replicates for each treatment. Data were processed as reported in our previous study [17]. Briefly, raw reads were first trimmed adapters and removed low-quality bases using Trimmomatic version 0.36 [25], aligned to *Oryza sativa* spp. *Japonica* reference genome International Rice Genome Sequencing Project (IRGSP)-1.0; [26, 27] using HISAT2 version 2.1.0 [28]. StringTie v1.3.4d [29] was used to assemble the transcripts within the regions and obtain the gene counts.

### Differential expression, gene ontology enrichment, and KEGG pathway analyses

Differential expression analysis was performed in R studio using DESeq2 v1.26 [30] with reads normalizing and variance stabilizing transformation to account for library size and sequencing depth differences. For this study a generalized linear model was used:

$$Y=\tau_{Trt}+\gamma_{MCP}+\tau_{Trt}\times \gamma_{MCP}$$

where *Y* is the read count for each gene, with three explanatory variables, including heat treatment (HNT and ANT), 1-MCP treatment (1-MCP and without 1-MCP), and interaction between heat treatment and 1-MCP treatment, was used in this study. Due to the genetic background differences between Antonio and Colorado, data from the two varieties were fitted in the model separately. Internally, *p*-values were adjusted for multiple testing using the Benjamini–Hochberg method in the DESeq2 package. Genes with a false discovery rate (FDR) adjusted *P*-value (*P*_adj_) < 0.05 were identified as differentially expressed genes (DEGs).

There were five DEG lists used for the gene ontology (GO) enrichment and Kyoto Encyclopedia of Genes and Genomes (KEGG) pathway analyses in each variety, including interaction effects between heat treatment and 1-MCP, heat effect without 1-MCP, heat effect with 1-MCP, 1-MCP effect under ANT, and 1-MCP effect under HNT. Gene ontology enrichment analysis and KEGG pathway analysis were performed using the web-based tool g:Profiler (https://biit.cs.ut.ee/gprofiler/gost) [31]. The significant threshold of GO and KEGG analyses was determined by Benjamini–Hochberg method with FDR adjusted. GO terms and KEGG pathways with FDR < 0.05 were identified as significant.

### Protein–protein interaction (PPI) network analysis and hub gene identification

In order to understand the potential interaction between identified DEGs, protein–protein interaction (PPI) network analysis was performed based on the STRING database (https://string-db.org) [32]. The PPI networks were constructed and visualized using StringApp v.1.7.0 [33] on Cytoscape software v.3.9.0 [34]. To explore the potential connected regions of the network, "full STRING network" was used in StringApp, with default setting of an interaction score cutoff of 0.4 and a maximum additional interactors of 0; and protein sources were limited to *Oryza sativa*. The resulting PPI network was then analyzed for hub genes using cytoHubba [35]. In our study, hub genes were defined by top 20 nodes ranked by maximal clique centrality (MCC) score.

## Results and discussion

### Physiological response under high-time temperature (HNT)

The results of the current study indicated differential physiological responses to HNT and 1-MCP with respect to yield, spikelet fertility, and leaf photosynthetic rate (Table 1). Under HNT condition, the responses among the three physiological characteristics in Antonio had significantly decreased, ranging from 20–55% (Table 1). On the other hand, Colorado had reduced spikelet fertility and leaf photosynthetic rate (11% and 9%, respectively), but there was no significant decrease in yield (Table 1). Taken together, Colorado was less affected by HNT than Antonio. Previous studies have reported reduced spikelet fertility, photosynthetic rate, and yield as a result of HNT [6, 14, 36]. The decreased spikelet fertility is associated with impaired hormonal balance in the floret [37] and/or reduced availability of photosynthates to the kernel [38] and/or inability of floral buds to mobilize carbohydrates under heat stress [39].

**Table 1:** Effect of variety on physiological traits under ambient night-time temperature (ANT) and high night-time temperature (HNT) conditions, with or without MCP (1-methylcyclopropene) treatment for the heat susceptible variety, Antonio and heat tolerant variety, Colorado.

Traits	ANT^1^		HNT^1^		Effects of HNT (%)^2^		ANT-MCP		Effects of MCP.ANT (%)^3^		HNT-MCP		Effects of MCP.HNT^5^ (%)	
	A^4^	C^4^	A	C	A	C	A	C	A	C	A	C	A	C
Yield (g plant^-1^)	9.7	9.9	4.4	10.7	-55	NS	10.3	9.1	NS	NS	11.2	9.6	156	NS
Spikelet fertility (%)	79.7	81.9	36.9	72.9	-54	-11	70.8	78.2	-11	NS	71.9	62.7	95	-14
Leaf photosynthetic rate (umol m^-2^s^-1^)	20.5	21.4	16.3	19.5	-20	-9	19.5	21.1	NS	NS	18.0	20.9	10	NS

^1^ANT (25°C; 77°F); HNT (30°C; 86°F)

^2^Effects of HNT (%): This is the percent difference of the effects due to HNT, where a negative % corresponds to harmful effects.

^3^Effects of MCP at ANT (%): This indicates the effects of 1-MCP under ANT conditions compared to the control (no 1-MCP treatment) under ANT.

^4^The “A” indicates the heat susceptible variety, Antonio, whereas the “C” indicates the heat tolerant variety Colorado.

^5^Effects of MCP at HNT (%), where a positive % corresponds to a beneficial effect.

To understand whether 1-MCP can mitigate the negative effects of HNT and the response of two rice varieties under ANT, 1-MCP was applied to the two rice varieties undergoing ANT and HNT treatments. The results showed that the application of 1-MCP significantly prevented negative effects of HNT in Antonio (Table 1). In contrast, there were no significant differences for yield and leaf photosynthetic rate in Colorado (Table 1). Interestingly, 1-MCP may provide a slightly negative effect on spikelet fertility in Colorado under HNT (Table 1). Under ANT conditions, 1-MCP did not show any significant change in physiological response among traits except an 11% reduction in spikelet fertility for Antonio (Table 1). Previous studies also reported the beneficial effects of the 1-MCP application under heat stress in rice and soybean [7, 14].

### Principal component analysis of RNA-seq samples and overview of DEGs

There were three explanatory variables evaluated in the current study, which include a variety (Antonio: heat susceptible and Colorado: heat tolerant) variable, heat (HNT and ANT), and 1-MCP treatment (1-MCP and without 1-MCP). To explore the comprehensive gene expression differences of the HNT susceptible variety, Antonio, and the tolerant variety, Colorado, under HNT conditions and with and without the 1-MCP treatment, a total of 38,909 annotated rice genes with at least 10 counts were used for principal component (PCA) analysis. As sources of variation were explored, variety appeared to be the most significant factor in explaining the variations in gene expression and explained 47% of the total variance (see S1 Fig). Samples from the same genotype grouped close together regardless of the HNT and 1-MCP conditions, indicating that samples with the same genotype had relatively similar gene expression patterns. For the HNT tolerant variety, Colorado, the second most important factor was HNT treatment, which clearly separated samples into two groups regardless of the 1-MCP application. On the other hand, the susceptible variety, Antonio, showed that both HNT and ANT samples had similar gene expressions without 1-MCP application. However, with 1-MCP application, HNT and ANT samples displayed separately as in the tolerant variety Colorado.

**Fig 1:**
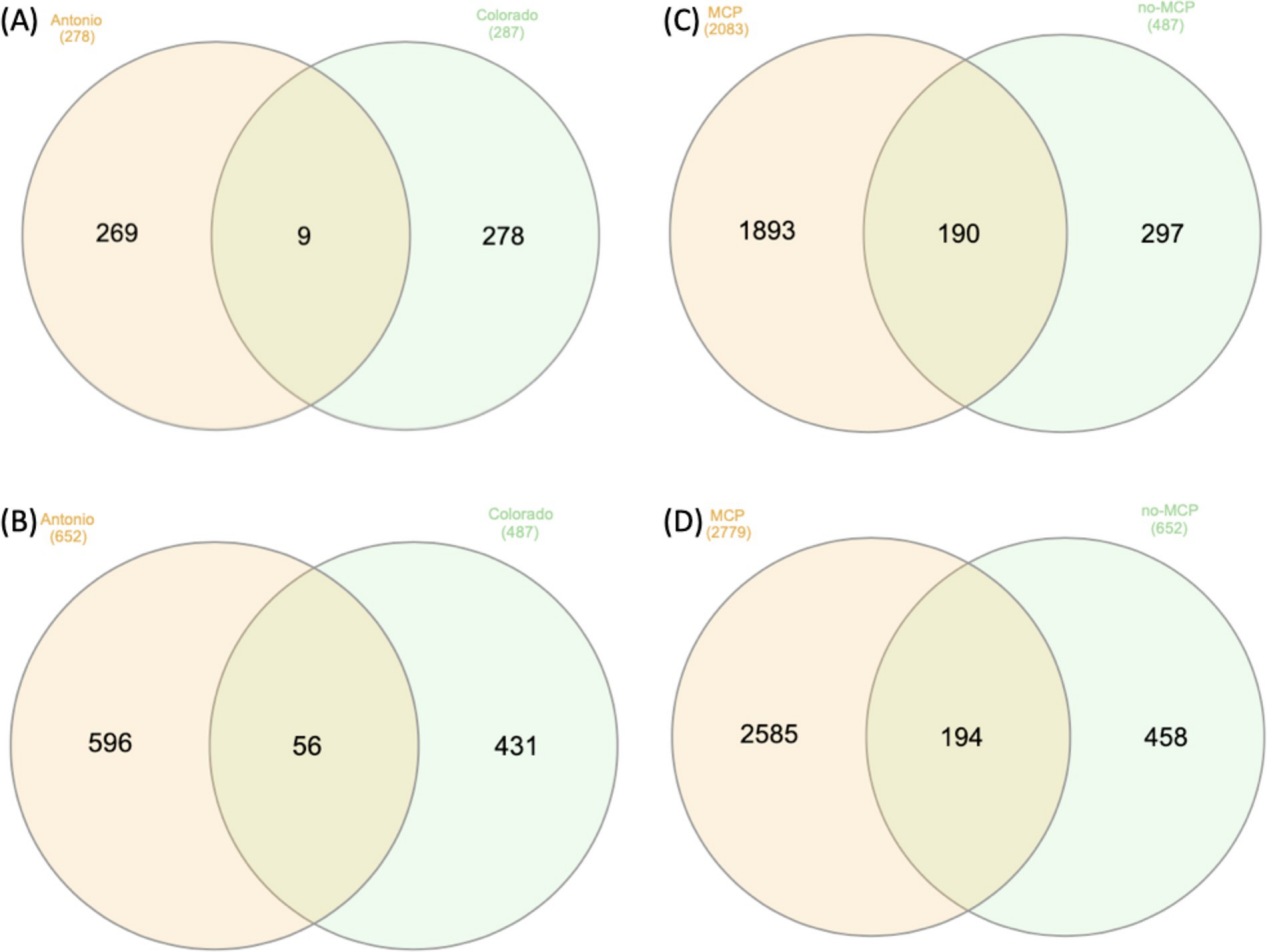
Venn diagrams of the number of DEGs between each comparison. (A) HNT tolerant genotype Colorado and susceptible genotype Antonio after MCP application under control condition (ANT). (B) HNT responsive DEGs in Antonio and Colorado without MCP application. (C) DEGs between with and without MCP application in Colorado under HNT condition. (D) DEGs between with and without MCP application in Antonio under HNT condition.

To minimize the effect of different genetic backgrounds, data from Antonio and Colorado were analyzed independently. Venn diagrams illustrated the number of DEGs for different comparisons (Fig 1). A total of 278 and 287 genes were identified to be differentially expressed after 1-MCP application under ANT conditions for Antonio and Colorado, respectively (Fig 1A). Among all of the 1-MCP responsive DEGs, only nine DEGs were common between the two varieties (Table 2), suggesting that the varieties had very distinct responses toward 1-MCP. Under HNT conditions, Antonio had 652 DEGs and Colorado had 487 DEGs. There were 56 DEGs commonly identified in both varieties (Fig 1B). These 56 common DEGs were enriched in only one molecular function GO term (GO:0016165)- linoleate 13S-lipoxygenase activity, which can be related to the lipid peroxidation after heat stress. Previous studies showed that lipoxygenase (LOX) plays an important role in fatty acid oxidation and can cause membrane degradation under stress [40–42]. The results suggested that the two varieties had responded to heat stress and had very distinct responsive mechanisms. In addition, under HNT conditions, both Antonio and Colorado had four times more DEGs with 1-MCP compared to those without 1-MCP (Fig 1C and 1D).

**Table 2:** Common DEGs between Antonio and Colorado after MCP application under control condition.

Gene ID	Log2-fold-change		Description
	Antonio	Colorado	
*Os01g0342500*	0.77	1.06	Hypothetical conserved gene
*Os01g0803200*	0.62	0.52	Cysteine proteinase inhibitor-I
*Os02g0809800*	-2.07	-1.41	Root-to-shoot Phosphate (Pi) transporter
*Os03g0170900*	-0.78	-1.13	Sucrose transporter
*Os05g0165900*	0.98	-1.12	S-Domain receptor like kinase-34
*Os06g0244000*	-3.63	-3.95	Similar to anthranilic acid methyltransferase 3
*Os07g0634400*	-2.29	-2.25	HAD-superfamily hydrolase
*Os09g0494600*	-2.72	-1.50	Protein of unknown function DUF599 family protein
*Os11g0115400*	0.42	1.09	Lipid transfer protein

### Molecular response to high night-time temperature (HNT)

Under HNT conditions, Antonio had 652 DEGs and Colorado had 487 DEGs without 1-MCP application (Fig 1B; S1 Table). In Antonio, there were 215 DEGs that up-regulated and 437 DEGs that were down-regulated. On the other hand, Colorado had 102 DEGs that were up-regulated and 385 DEGs that were down-regulated. For both varieties, the top two largely up-regulated DEGs were unannotated genes, including *Os03g0232050* and *Os08g0327501* in Antonio, and *Os01g0234001* and *Os03g0221600* in Colorado (S1 Table). To understand the potential function of these DEGs, their sequences were BLAST against NCBI database. The results showed that *Os03g0232050* is perfectly matched for predicted F-box protein SKIP22 (NCBI sequence ID: XM_015775845.2) and *Os03g0221600* is perfectly matched for predicted G-type lectin S-receptor-like serine/threonine-protein kinase (NCBI sequence ID: XM_015775926.2). The F-box proteins have been linked to stress response and ethylene-signaling pathway [43]. Previous studies reported that F-box proteins have positive function in wheat [44, 45] but possibly have a negative effect on abiotic stress tolerance in rice [46]. To our knowledge, there is no reported molecular work in rice for lectin S-receptor-like kinase (RLK); however, based on the studies in wheat, *Arabidopsis*, and tomato, RLKs have a function related to salt tolerance, plasma membrane stability, and disease resistance [47–50]. In addition, there were three photosynthesis-related genes that were down-regulated in Antonio, including Photosystem II protein Psb28 (*Os01g0938100*), 22-kDa Photosystem II protein (*Os01g0869800*), and Photosystem I PsaO domain-containing protein (*Os04g0414700*). On the other hand, no photosynthesis related DEGs were identified in Colorado.

To understand the functional classification underlying these DEGs, GO enrichment and KEGG enrichment analyses were performed. There were 19 GO terms enriched in Antonio in response to HNT, including 14 GO terms in biological process (BP), such as chromatin assembly, DNA packaging, oxoacid metabolic process, response to heat and so on; one GO term enriched in molecular function (MF); and four GO terms enriched in cellular component (CC) (S2 Table). Interestingly, there were three KEGG pathways enriched in Antonio, biosynthesis of secondary metabolites, nitrogen metabolism, and arginine and proline metabolism. For Colorado, there were eight BP terms, two MF terms, and two CC terms enriched. There was no stress-related GO term that was enriched in Colorado, but there was an enriched KEGG term (KEGG:00902 Monoterpenoid biosynthesis) linked to a stress response. DEGs that are involved in Monoterpenoid biosynthesis pathway, including short-chain dehydrogenase/reductase (SDR) domain containing protein (*Os04g0531900* and *Os04g0532100*) and similar to salutaridine reductase protein (*Os04g0532400*). SDR proteins form one of the largest and oldest NAD(P)(H)- dependent oxidoreductase families and have been demonstrated in a variety of primary and secondary metabolisms, including lipid synthesis and chlorophyll biosynthesis or degradation [51, 52]. These DEGs were down-regulated in Colorado, while there were no expression differences in Antonio. Taken together, our findings suggest that Colorado is less sensitive to HNT, therefore with no significant response to the stress.

### Molecular response to 1-MCP treatment under ambient night-time temperature (ANT)

Results of physiological traits showed that 1-MCP did not cause any negative effect under ANT conditions for both varieties, except for spikelet fertility which decreased by 11% in Antonio (Table 1). To understand how 1-MCP affects transcriptional regulation mechanisms, we first looked at the different gene expressions of the two varieties under ANT conditions. Among the 278 DEGs in Antonio with 1-MCP and without 1-MCP treatment, there were three GO terms and three KEGG pathways enriched, including auxin-activated signaling pathway (GO:0009734), cellular response to auxin stimulus (GO:0071365), plasma membrane (GO:0005886), carbon fixation in photosynthetic organisms (KEGG:00710), nitrogen metabolism (KEGG:00910), and glyoxylate and dicarboxylate metabolism (KEGG:00630) (S3 Table). In Colorado there were 39 GO terms enriched among the 287 DEGs including, eight MF terms, 22 BP terms, nine CC terms, and one KEGG pathway. The majority of the enriched GO terms and KEGG pathway were with structure-related functions such as DNA-binding, chromatin assembly, protein-complex, and ribosome (S4 Table). In contrast to the results in Antonio, there was no hormone signaling-related terms enriched in Colorado.

It is known that 1-MCP works as an ethylene perception inhibitor, therefore, we looked into the DEGs list of the two varieties to see whether there were any ethylene-related genes identified. The results showed that anther ethylene-upregulated protein ER1 (*Os03g0388500*) had an increased expression level, and ethylene response factor 2 (*Os09g0434500*) had expression level decreased in Antonio. There were no ethylene-related DEGs identified in Colorado (S4 Table). Interestingly, we found two ethylene-related genes, ACC oxidase, ethylene biosynthesis (*Os05g0149400*) and similar to anther ethylene-upregulated protein ER1 (*Os03g0388500*) were down-regulated in Colorado under HNT without 1-MCP treatment. On the other hand, *Os03g0388500* was up-regulated in Antonio (S1 Table). Taken together we hypothesize that Colorado may be less sensitive to ethylene or has less ethylene accumulated under HNT. With 1-MCP application, there were only nine DEGs commonly identified within two varieties (Fig 1A). The common DEG list showed that all the DEGs maintained the same trend in two varieties except for S-Domain receptor-like kinase-34 (*Os05g0165900*), which had expression increased in Antonio but decreased in Colorado (Table 2).

### Molecular response of 1-MCP regulates HNT response

**Fig 2:**
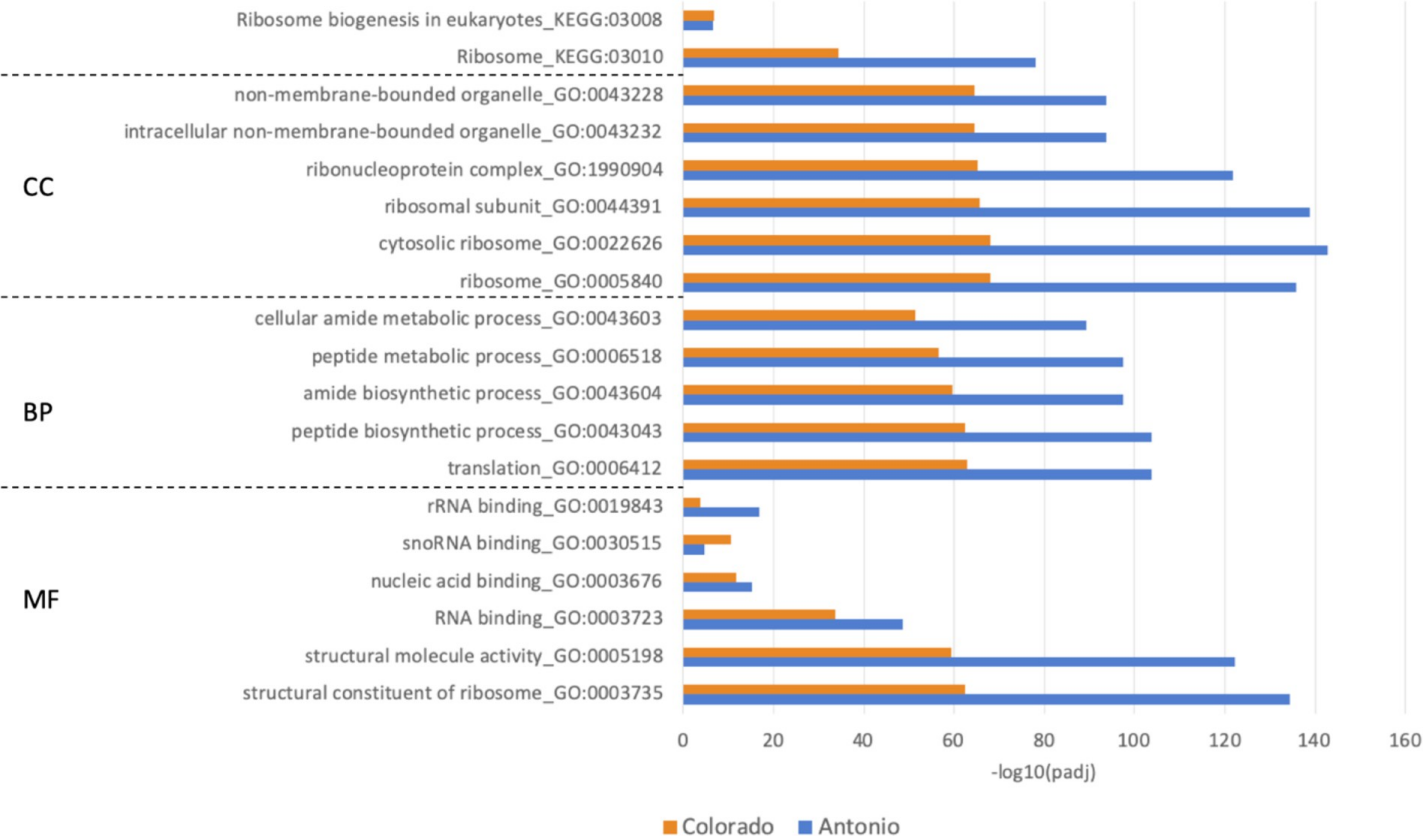
Top five enriched GO terms in Antonio and Colorado under HNT with MCP application.

The results showed that 1-MCP induced more DEGs compared to those without 1-MCP application (Fig 1C and 1D). Hence, we first examined 1-MCP effect under HNT condition (i.e. identifying DEGs between with and without 1-MCP). There were 683 and 438 DEGs identified in Antonio and Colorado, respectively (S5 Table), with 131 common DEGs identified in both varieties. The results from enrichment analysis showed that DEGs from the two varieties were enriched in similar functional groups (S6 Table). The top five GO terms and KEGG pathways, were selected based on having the smallest adjusted p-values. Our results showed that the two varieties shared the same top five GO terms in BP and four GO terms with MF and CC (Fig 2). Majority of these GO terms and pathways were associated with translational regulation and structure. The only significant enriched GO term related to hormone signaling was cytokinin-activated signaling pathway (GO:0009736) in Antonio (S6 Table). The DEG lists of the two varieties showed that Antonio had several ethylene-related and transcription factor (TF) DEGs, while Colorado had no ethylene-related DEGs (Table 3; S5 Table). There were two ethylene biosynthesis genes, ACC Oxidase (*Os09g0451000* and *Os09g0451400*) primarily up-regulated in Antonio (Table 3). Notably, these two ethylene biosynthesis genes were different from Anther ethylene-upregulated protein ER1 (*Os03g0388500*), which were up-regulated under HNT in Antonio. In addition, ethylene-responsive transcriptional coactivator (*Os06g0592500*) was the only commonly transcriptional regulator identified in both varieties and it was up-regulated in both. Our results also showed that that the heat stress transcription factor B-2b (*Os08g0546800*) was up-regulated in Antonio but not in Colorado. In addition, a MADS-box transcription factor (*Os03g0753100*) was largely up-regulated solely in Antonio (Table 3). *Os03g0753100* has been previously reported to be related with inflorescence and spikelet development [53]. These expression-level differences may partly explain why 1-MCP only improved HNT tolerance of Antonio but not Colorado.

**Table 3:** The expression differences of ethylene-related genes and transcription factors caused by MCP effect under HNT.

		Log2-fold-change	
Gene ID	Description	Antonio	Colorado
*Os01g0235700*	Similar to BHLH transcription factor (Fragment).	1.24	ns
*Os01g0948200*	Similar to GRAS family transcription factor containing protein.	-1.03	ns
*Os01g0976800*	GATA transcription factor, Regulation of fertility conversion of two-line hybrid tms5 mutant rice	-1.01	ns
*Os03g0161900*	Similar to Isoform 2 of Heat stress transcription factor A-2d.	0.62	ns
*Os03g0327800*	NAC Family transcriptional activator, Abiotic stress response, Positive regulator of leaf senescence	-1.08	ns
*Os03g0650000*	YABBY transcription factor, Control of seed shattering	1.84	ns
*Os03g0753100*	MADS-box transcription factor, Inflorescence and spikelet development	3.18	ns
*Os03g0838900*	Mitochodrial transcription termination factor-related domain containing protein.	ns	-0.53
*Os03g0851000*	Similar to Transcription factor homolog BTF3-like protein.	-0.68	ns
*Os04g0546800*	Pathogenesis-related transcriptional factor and ERF domain containing protein.	-0.86	ns
*Os06g0592500*	Similar to Ethylene-responsive transcriptional coactivator.	1.57	1.41
*Os07g0679700*	Transcriptional factor B3 family protein.	ns	0.55
*Os07g0686100*	Drought stress-related bZIP transcription factor, Positive regulation of drought and oxidative tolerance	ns	-1.02
*Os08g0546800*	Similar to Heat stress transcription factor B-2b.	1.00	ns
*Os09g0114500*	Kinesin-4 protein with transcription regulation activity, Cell cycle and wall modification, Cell elongation by regulating GA biosynthesis pathway	0.48	ns
*Os09g0451000*	ACC oxidase, Ethylene biosynthesis	1.03	ns
*Os09g0451400*	ACC oxidase, Ethylene biosynthesis	8.81	ns
*Os10g0369600*	Similar to GRAS transcription factor (Fragment).	ns	-0.45
*Os11g0558200*	R2R3-type MYB transcriptional activator, Tolerance to Pi deficiency	-0.62	ns
*Os12g0610600*	NAC transcription factor, Negative regulation of drought tolerance	-1.10	ns

We then investigated how HNT affects gene expression, while 1-MCP was applied to both HNT and ANT conditions. Comparing ANT and HNT, with 1-MCP application compared to without, the number of DEGs were three times higher for both Antonio and Colorado (Fig 1C and 1D). There were 2779 and 2083 DEGs identified in Antonio and Colorado, respectively (S7 Table), while there were 652 and 487 without the application of 1-MCP. Among the identified DEGs in two varieties, 600 of them were commonly identified, including eleven heat shock proteins (HSPs) (S7 Table). We also found that the heat stress associated protein (*Os06g0682900*) was up-regulated in both varieties, but it was largely increased in Antonio, with log2-fold-change of 1.46, comparing to 0.79 in Colorado. Interestingly, *Os06g0682900* was not identified as a DEG under HNT without 1-MCP (S1 Table). In agreement with the results above, GO and KEGG enrichment analyses showed that DEGs in both varieties were strongly associated with translational regulation and structure, except there were several GO terms related to hydrolase activity enriched in Colorado (S7 Table). Focusing on ethylene and TF-related DEGs, we found that Antonio and Colorado had very distinct DEG profiles. Although both Antonio and Colorado had several down-regulated ethylene-responsive transcription factors, they were not the same (Table 4). The only DEG identified in both varieties was ACC oxidase, ethylene biosynthesis (*Os05g0149400*), which was down-regulated in both varieties. However, the results also showed that there were another two ACC oxidase, ethylene biosynthesis genes (*Os09g0451000* and *Os09g0451000*) up-regulated in Antonio (Table 4). The up-regulated ethylene biosynthesis genes could be responding to the lower sensitivity of ethylene response in Antonio after the application of 1-MCP. Blocking ethylene perception during crop growth can also prevent the abscission of leaves and flowers and the yellowing of vegetables [43].

**Table 4:** The expression differences of ethylene-related genes and transcription factors of HNT effect with MCP application.

		Log2-fold-change	
Gene ID	Description	Antonio	Colorado
*Os03g0182800*	Similar to Ethylene responsive element binding factor3 (OsERF3).	ns	-2.26
*Os03g0183000*	APETALA2/ethylene-responsive element binding protein 125, Transcription regulator, Positive regulator of grain length	-1.35	ns
*Os03g0183300*	Pathogenesis-related transcriptional factor and ERF domain containing protein.	-0.59	ns
*Os03g0388500*	Similar to Anther ethylene-upregulated protein ER1 (Fragment).	ns	-0.75
*Os04g0546800*	Pathogenesis-related transcriptional factor and ERF domain containing protein.	-1.60	ns
*Os05g0149400*	ACC oxidase, Ethylene biosynthesis	-1.13	-0.98
*Os09g0286600*	Pathogenesis-related transcriptional factor and ERF domain containing protein.	-0.73	ns
*Os09g0287000*	Similar to Ethylene-responsive transcription factor 5 (Ethylene-responsive element binding factor 5) (EREBP-5) (AtERF5).	-1.85	ns
*Os05g0316800*	Similar to Ethylene-responsive transcription factor 9 (Ethylene-responsive element binding factor 9) (EREBP-9) (AtERF9).	-0.93	ns
*Os09g0434500*	Similar to Ethylene response factor 2.	ns	0.90
*Os09g0451000*	ACC oxidase, Ethylene biosynthesis	1.17	ns
*Os09g0451400*	ACC oxidase, Ethylene biosynthesis	2.28	ns

### Expression patterns of genes involved in ethylene perception

The results above showed that Antonio and Colorado had distinct responsive mechanisms in response to both HNT and 1-MCP. In addition, some genes had different expression patterns under the interaction between HNT and 1-MCP treatments. Therefore, we examined the DEGs regulated by the HNT and 1-MCP interaction effects. The results showed that 1106 DEGs in Antonio responded differently under different conditions, while only 482 DEGs in Colorado behaved like that (S8 Table). Further, we found that there were several TFs, stress and/or ethylene-related DEGs uniquely identified in Antonio, including ethylene-responsive transcriptional coactivator (*Os06g0592500*), several ethylene response factors (ERF) domain containing protein (*Os01g0693400*, *Os04g0529100*, *Os04g0546800*, *Os06g0592500*), MADS-box TF (*Os03g0752800*, *Os06g0108500*, *Os07g0108900*, *Os08g0112700*), and senescence-associated and/or NAM, ATAF and NAC TF (*Os03g0327800*, *Os08g0433500*, *Os08g0490100*, *Os12g0610600*). Among all TFs, only MADS-box transcription factor 15 (*Os07g0108900*) and regulator for phosphate homeostasis (*Os04g0671900*) were also identified in Colorado. These ethylene and senescence-related TFs were only identified in Antonio, suggesting that 1-MCP affected ethylene signaling pathway in Antonio but not Colorado (S8 Table).

**Fig 3:**
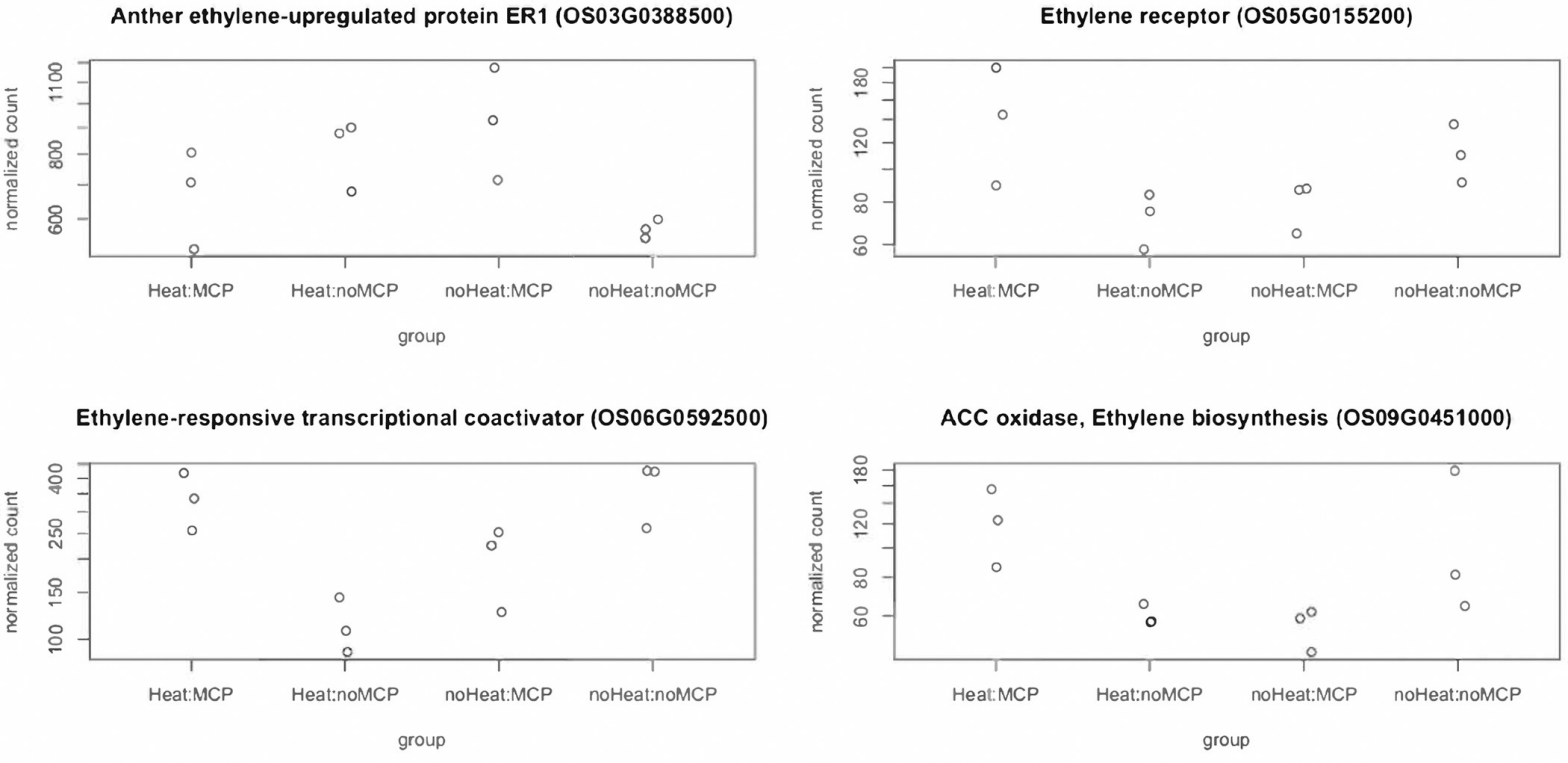
Ethylene-related differentially expressed genes (DEGs) under 1-MCP treatment and HNT interaction effect in Antonio.

**Fig 4:**
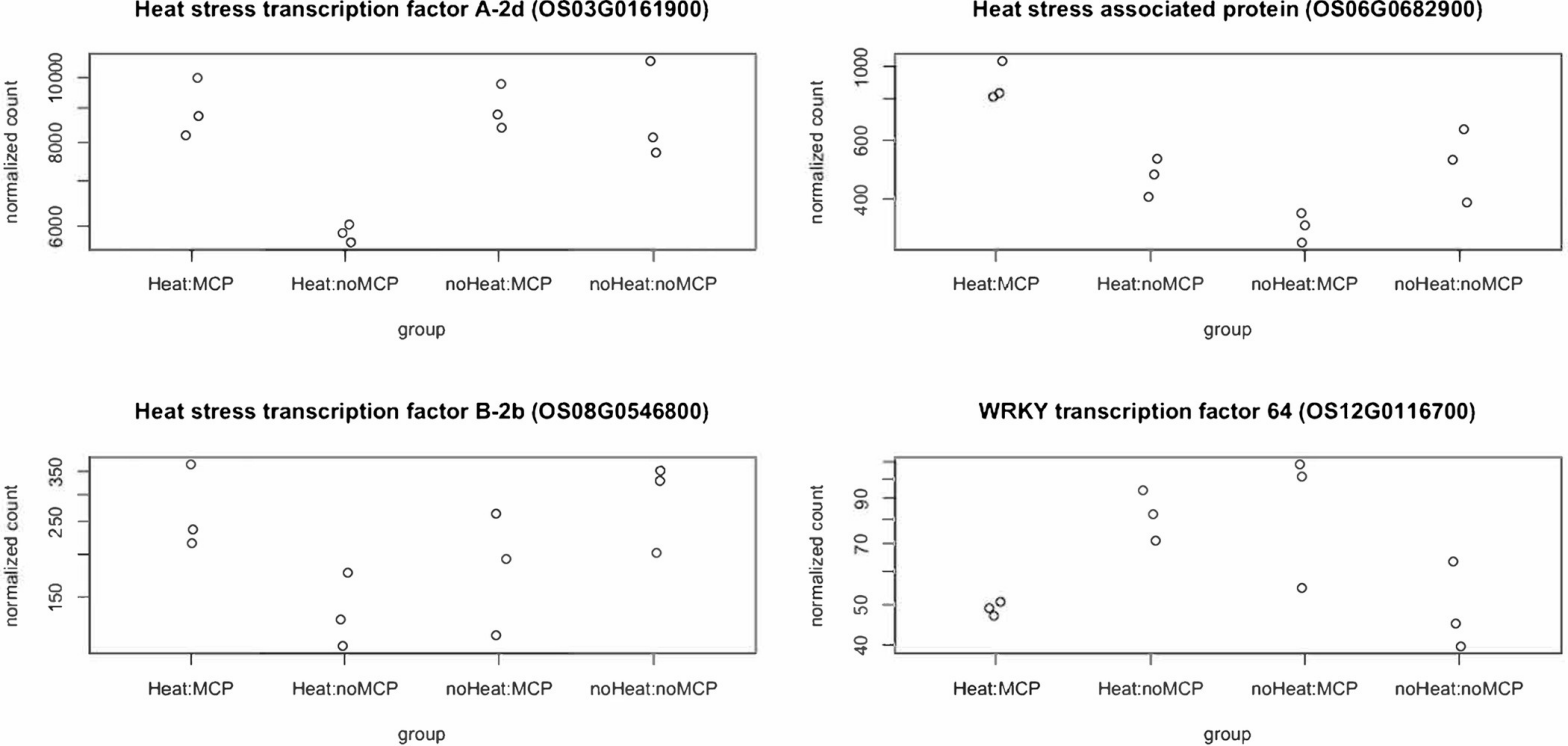
Heat and stress-related transcription factors under 1-MCP and HNT interaction effect in Antonio.

We then looked into ethylene-related DEGs and examined the normalized read count. The results showed that these genes had similar expression level between ANT without 1-MCP group and HNT with 1-MCP group. For example, ethylene receptor (*Os05g0155200*) had similar expression level in “Heat:noMCP” and “noHeat:MCP” groups, whereas “Heat:MCP” had similar level with “noHeat:noMCP” (Fig 3). These results demonstrated how 1-MCP mitigates HNT effects in Antonio through the ethylene-related regulation and allowed the plant to have perception as ANT condition. We then looked into heat stress and other stress-related TFs that responded to the interaction between HNT and 1-MCP. There were two expression patterns for these genes. First, heat stress transcription factor A-2d (*Os03g0161900*) had lower expression level in “Heat:noMCP” but with much higher expression level in the rest three conditions (Fig 4). The second pattern is that gene expressed similarly in “Heat:MCP” and “noHeat:noMCP”, vice versa. For instance, heat stress associated protein (*Os06g0682900*) had a similar pattern in “Heat:MCP” and “noHeat:noMCP” groups, while “Heat:MCP” had the highest expression level among all four combinations. There was one TF that showed higher expression level in “Heat:noMCP” and “noHeat:MCP” groups- WRKY TF64 (*Os12g0116700*). The WRKY family proteins are a class of plant-specific TFs that are involved in several stress response pathways [54, 55]. It has been reported previously in rice that these proteins were mainly involved with pathogen defense regulation. However, based on our results, WRKY TF64 may play a role as a negative regulator in response to HNT in Antonio. Another thing to note is that the response mechanism of HNT condition may be different from commonly defined heat stress. Common heat stress occurs during both daytime and night-time and has significant impact on membrane dysfunction, protein denaturation, nucleotide damage, and changes in lipid metabolism [13, 56]. On the contrary, other than accumulation of ethylene, HNT condition can also increase the daytime leaf photosynthetic rates (Pn) in the following day by reducing carbohydrate-induced feedback inhibition of photosynthesis [6, 57].

Ethylene is known to play significant roles in plant growth regulation and overall resilience to stress. For plant growth regulation, ethylene plays dual roles in both promoting and inhibiting growth depending on the species and concentration. For instance, it inhibits primary root and leaf growth, but it can also promote the growth of young leaves in the presence of very low ethylene concentrations in bluegrass and sunflowers [58]. Ethylene biosynthesis genes promote ethylene accumulation in roots, which inhibits root elongation and promotes the formation of shallow roots. While more shallow roots may help plants survive under stress, they are not favorable for the uptake of water and nitrogen, therefore frequently resulting in decreased yield [59]. In rice, ethylene’s impact on stress responses is also mediated through its interactions with other plant hormones, such as auxin, jasmonic acid, gibberellin, and abscisic acid [60], as well as downstream signaling pathways such as CBF/DREB1 (C-repeat binding factor/dehydration-responsive element-binding factor) [61], which influence both short-term stress responses and long-term developmental outcomes. For example, the Submergence 1 (Sub1), an ERF TF-encoding gene, enhances the plant’s tolerance to submergence during flooding by suppressing the synthesis and signaling of gibberellin, which helps to restrict unnecessary growth and elongation, conserving energy and enabling the rice plants to survive longer periods of submergence [62]. A previous study revealed that when ethylene binding inhibitors, such as 1-MCP and silver nitrate, were applied as foliar sprays, both abscission and yellowing were diminished. Another study also showed that changes to the ethylene signaling pathway affect rice grain filling and grain size [63]. Therefore, we hypothesize that the improved performance of Antonio under HNT with the application of 1-MCP was due to the inhibition of ethylene response pathways. Further studies, such as precisely knocking out or regulating the expression levels of individual DEGs using CRISPR gene editing, would be highly beneficial to explore the mechanisms of HNT response, with or without the application of 1-MCP. Additionally, a detailed comparative genomic analysis between the two rice varieties would also be advantageous for narrowing down potential targets for future breeding programs.

### Identification of hub genes

**Fig 5:**
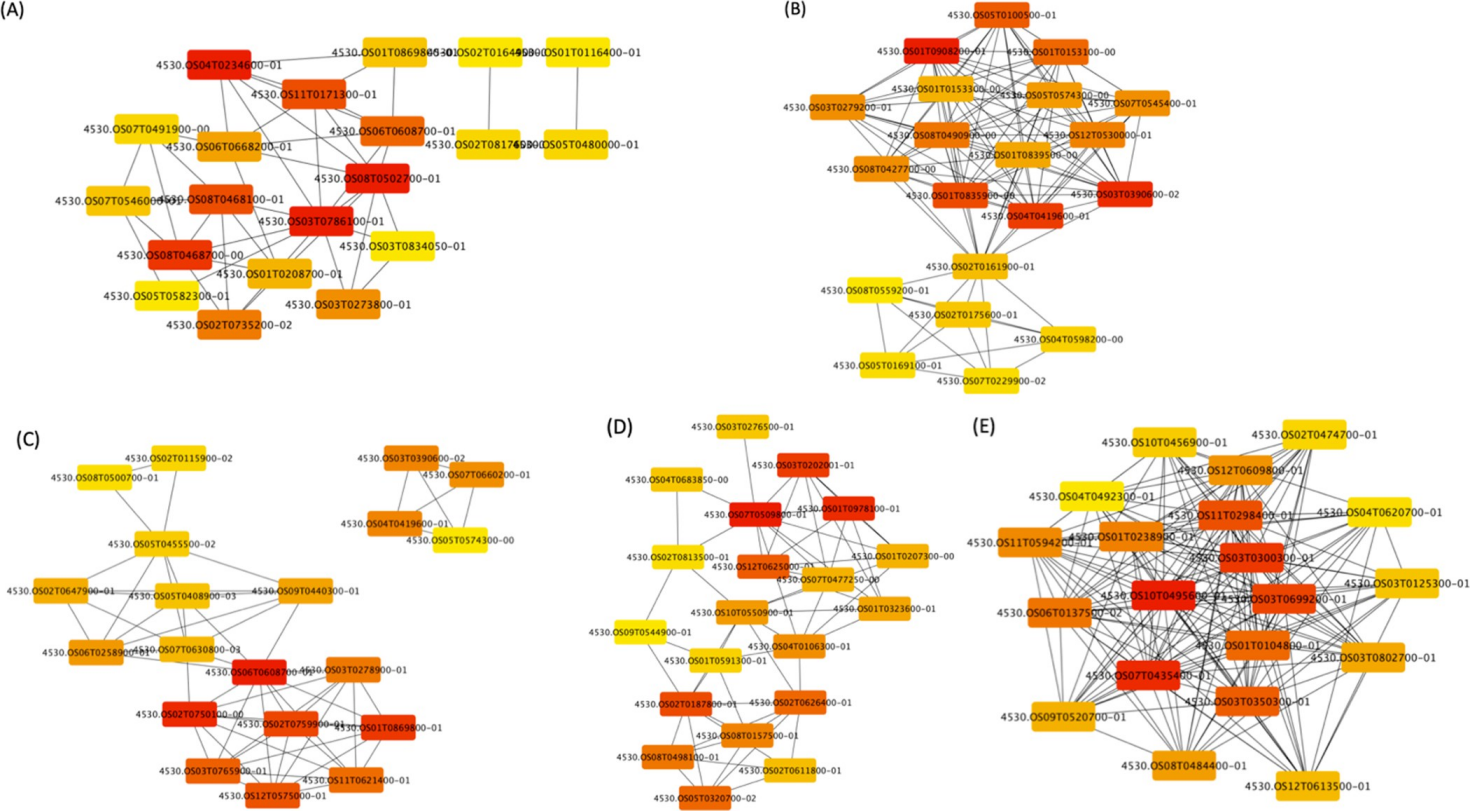
Hub genes identified from DEGs of (A) Antonio ANT x ANT-MCP, (B) Colorado ANT x ANT-MCP, (C) Antonio ANT x HNT, (D) Colorado ANT x HNT, and (E) Colorado Trt x MCP. Red color indicates a higher MCC score, meaning a more important hub gene.

The top 20 hub genes were identified from DEGs of different comparisons (Fig 5; S9 Table). Due to the complex relationship between DEGs, some networks of Antonio comparisons were unable to be computed. However, the night-time temperature of 25°C has been considered as mild stress (optimum temperature is 21°C; A.R. Mohammed and L. Tarpley, unpublished). Therefore, we first looked into hub genes identified in DEG of 1-MCP treatment under 25°C. For Antonio, KEGG analysis suggested that the hub genes are enriched in Carbon fixation in photosynthetic organisms, Nitrogen metabolism, Carbon metabolism, and Metabolic pathways. On the other hand, the only enriched KEGG pathway in Colorado is Ribosome. Moreover, the top hub gene in Antonio (*Os03g0786100)* is glycolate oxidase, which has strong regulation over photosynthesis. A previous study showed that overexpressed *Os03g0786100* improved photosynthesis and high light and high temperature response in rice [64]. No common hub genes were identified from DEGs of 30°C vs. 25°C for the two varieties. For Antonio, the hub genes are enriched in amino acid metabolism and degradation pathways. In contrast, Colorado has hub genes enriched in stress-responsive pathways including phenylpropanoid biosynthesis and biosynthesis of secondary metabolites. Phenylpropanoid biosynthesis has been reported to be related to plant defense mechanisms and stress tolerance regulation [65–68]. In particular, phenylpropanoids can enhance the production of photosynthetic pigments, nutrient uptake, and regulate growth. As for the hub genes identified from the interaction between temperature (ANT, HNT) and 1-MCP treatment, we noticed several of them are WD40-domain-containing genes. The WD40 proteins have been found to be involved in a wide range of cellular processes in plants including responses to abiotic interactions. For example, OsABT and SRWD proteins in rice and TaWD40D in wheat [69–71]. These results may explain why Colorado was less affected by HNT and why 1-MCP application largely improved the performance of Antonio under HNT.

## Conclusions

In summary, our physiological data suggested that Antonio is a HNT susceptible variety compared to Colorado. By investigating the transcriptomics data, we inferred that the HNT susceptibility of Antonio is largely affected by an increased ethylene-triggered metabolism. Therefore, with the application of 1-MCP, it mitigated the yield loss, the reduced spikelet fertility, and the decreased photosynthetic rate in Antonio. Future studies may explore the feasibility of 1-MCP applications at the field scale. Moreover, manipulation of key regulatory genes involved in the pathways identified in this study may assist in developing rice varieties more tolerant to HNT conditions, which will be increasingly critical to stabilize global rice production under the more extreme climate scenarios expected in the next few decades.

## Supporting information

S1 Fig Principal component analysis (PCA) of RNA-seq samples. Principal component analysis (PCA) of RNA-seq samples. The color differences indicate the different variety groups Antonio (orange) and Colorado (blue); the shape differences indicate no-HNT control (circles) and HNT treatment (triangles) groups; solid shapes indicate MCP treatment and translucent shapes indicate without MCP, as depicted in the figure legend on the right.
(PDF)

S1 Table Lists of differentially expressed genes under high night-time temperatures without 1-MCP application. (XLSX)

S2 Table Gene ontology (GO) enrichment analysis under high night-time temperatures without 1-MCP application. (XLSX)

S3 Table Lists of differentially expressed genes under ambient night-time temperatures with and without 1-MCP application. (XLSX)

S4 Table Gene ontology (GO) enrichment analysis under ambient night-time temperatures with and without 1-MCP application. (XLSX)

S5 Table Lists of differentially expressed genes under high night-time temperatures with and without 1-MCP application. (XLSX)

S6 Table Gene ontology (GO) enrichment analysis under high night-time temperatures with and without 1-MCP application. (XLSX)

S7 Table Lists of differentially expressed genes and gene ontology (GO) enrichment analysis under ambient versus high night-time temperatures with 1-MCP application. (XLSX)

S8 Table Lists of differentially expressed genes for the interaction between high night-time temperature and 1-MCP treatments. (XLSX)

S9 Table Lists of top 20 hub genes identified from differentially expressed genes (DEGs) between the different comparisons. (XLSX)
