# Supplementary material for: Transcriptome profiling of two rice varieties reveals their molecular responses under high night-time temperature
Source: PLoS One. 2024 Oct 10;19(10):e0311746. doi: 10.1371/journal.pone.0311746 (PMC11466396; doi:10.1371/journal.pone.0311746)
Supplement: S1 Fig — Principal component analysis (PCA) of RNA-seq samples. The color differences indicate the different variety groups Antonio (orange) and Colorado (blue); the shape differences indicate no-HNT control (circles) and HNT treatment (triangles) groups; solid shapes indicate MCP treatment and translucent shapes indicate without MCP, as depicted in the figure legend on the right. (PDF) [file pone.0311746.s001.pdf]

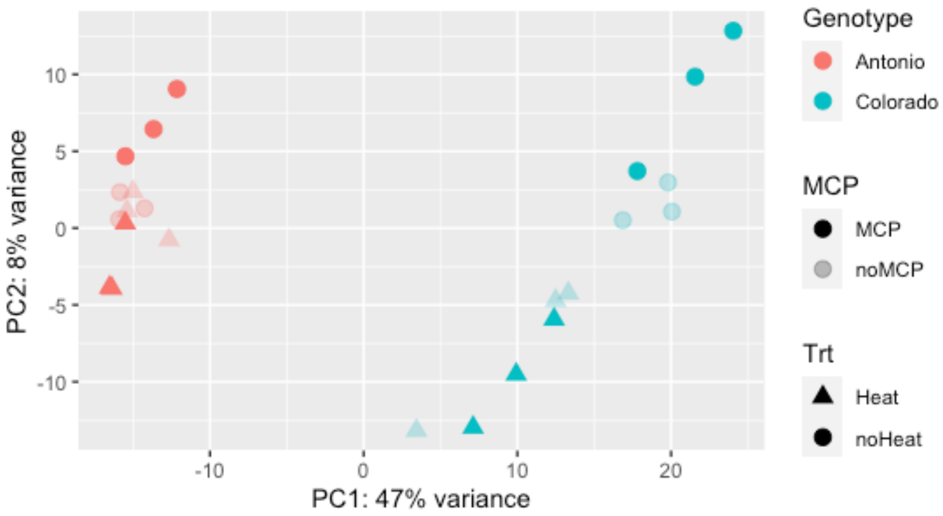

Supplementary Figure S1. Principal component analysis (PCA) of RNA-seq samples. The color differences indicate the different variety groups Antonio (orange) and Colorado (blue); the shape differences indicate no-HNT control (circles) and HNT treatment (triangles) groups; solid shapes indicate MCP treatment and translucent shapes indicate without MCP, as depicted in the figure legend on the right.
